# Promoted Growth and Multiband Emission in Heterostructured Perovskites Through Cs^+^‐Sublattice Interaction

**DOI:** 10.1002/advs.202306398

**Published:** 2023-11-28

**Authors:** Lei Zhou, Liangliang Liang, Jiaye Chen, Xin Zhou, Lingmei Liu, Shibo Xi, Kian Ping Loh, Yu Han, Qian He, Xiaogang Liu

**Affiliations:** ^1^ Department of Chemistry National University of Singapore Singapore 117549 Singapore; ^2^ School of Chemical Engineering and Technology Sun Yat‐sen University Zhuhai 519802 P. R. China; ^3^ Materials Science and Engineering National University of Singapore Singapore 117575 Singapore; ^4^ Multi‐scale Porous Materials Center Institute of Advanced Interdisciplinary Studies & School of Chemistry and Chemical Engineering Chongqing University Chongqing 400044 P. R. China; ^5^ Institute of Sustainability for Chemicals Energy and Environment (ISCE2) Agency for Science, Technology and Research (A*STAR) 1 Pesek Road Jurong Island Singapore 627833 Singapore; ^6^ Physical Sciences and Engineering Division Advanced Membranes and Porous Materials (AMPM) Center King Abdullah University of Science and Technology (KAUST) Thuwal 23955–6900 Saudi Arabia; ^7^ Institute of Materials Research and Engineering Agency for Science, Technology and Research Singapore 138634 Singapore; ^8^ The N1 Institute for Health National University of Singapore Singapore 117456 Singapore

**Keywords:** exciton confinement, growth kinetics, metal‐halide perovskite, metal‐organic framework, sublattice

## Abstract

Precise control of exciton confinement in metal halide perovskites is critical to the development of high‐performance, stable optoelectronic devices. A significant hurdle is the swift completion of ionic metathesis reactions, often within seconds, making consistent control challenging. Herein, the introduction of different steric hindrances in a Cs^+^ sublattice within CsYb_2_F_7_ is reported, which effectively modulates the reaction rate of Cs^+^ with lead (Pb^2+^) and halide ions in solution, extending the synthesis time for perovskite nanostructures to tens of minutes. Importantly, the Cs^+^ sublattice provides a crystal facet‐dependent preference for perovskite growth and thus exciton confinement, allowing the simultaneous occurrence of up to six emission bands of CsPbBr_3_. Moreover, the rigid CsYb_2_F_7_ nano template offers high activation energy and enhances the stability of the resulting perovskite nanostructures. This methodology provides a versatile approach to synthesizing functional heterostructures. Its robustness is demonstrated by in‐situ growth of perovskite nanostructures on Cs^+^‐mediated metal‐organic frameworks.

## Graphical abstract

Efficient steric hindrance management enables precise control of lead‐halide perovskite growth and emission from Cs^+^‐preconfined nanocrystals. Introducing diverse steric hindrances adjusts the Cs^+^ reaction rate with lead and halide ions in the solution, prolonging perovskite nanostructure synthesis to several tens of minutes. This approach has the potential to offer fresh perspectives in atomic‐scale nanocrystal engineering.

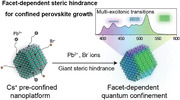

## 1 Introduction

Metal‐halide perovskite quantum dots (MHP QDs) have gained prominence in recent years because of their exceptional optical and electronic properties, including high luminescence quantum yield, narrow emission bandwidth, and tunable luminescence.^[^ ^1^ ^]^ However, the ionic nature of these compounds also poses several challenges to their synthesis and applications.^[^ ^2^ ^]^ Specifically, the nucleation and growth of MHP QDs occur too quickly through rapid ionic metathesis reactions, making it difficult to control with conventional hot injection or precipitation techniques. This poses a major obstacle to controlling the exciton confinement of QDs and a fundamental understanding of their nucleation, growth kinetics, and especially luminescence mechanisms.^[^ ^3^ ^]^ Despite enormous efforts, it remains challenging to contain the rapid growth of MHP QDs to achieve controlled exciton confinement.^[^ ^4^ ^]^ Unlike the widely used approach of using catalysts to lower the activation energy and thereby expedite organic synthesis, the introduction of steric hindrances can increase the activation energy and thus decelerate the reaction.^[^ ^5^ ^]^ Although surface engineering through ligands has proven effective in controlling the synthesis kinetics of perovskite QDs, organic ligands offer only a moderate steric hindrance.^[^ ^6^ ^]^

**Figure 1:**
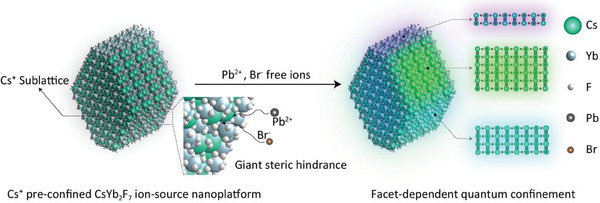
Controlling exciton confinement in lead halide perovskites using a Cs^+^‐preconfined nanoplatform. The enrichment of Cs^+^ ions in a robust crystalline framework provides a distinct steric hindrance, leading to a marked slowdown of the perovskite synthesis. The varied exposure of the Cs^+^ sublattice across distinct facets of the CsYb_2_F_7_ nanoplatform leads to facet‐dependent perovskite reconstruction and thus to significant variations in the exciton confinement of perovskite nanostructures.

Herein, we speculate that incorporating Cs^+^ ions into a rigid nanocrystal lattice might substantially reduce their reactivity toward other ions in solution, allowing subsequent control of the kinetics of perovskite growth on the surface. Moreover, the multifaceted nature of the selected nanocrystal matrix offers varied reaction environments, leading to facet‐specific reaction kinetics on a single nanoplatform. We reason that a rationally designed Cs^+^‐sublattice nanoplatform will allow perovskite nanostructures to be tailored from multilayers down to molecular dimensions, thereby achieving refined exciton confinement (**Figure** **1**). On a Cs^+^‐sublattice nanoplatform, we can observe fluorescence emissions stemming from various exciton confinements. In addition, the inherent rigidity of the Cs^+^‐sublattice suggests that the resulting perovskite nanostructures may exhibit enhanced stability.

## 2 Results and Discussion

**Figure 2:**
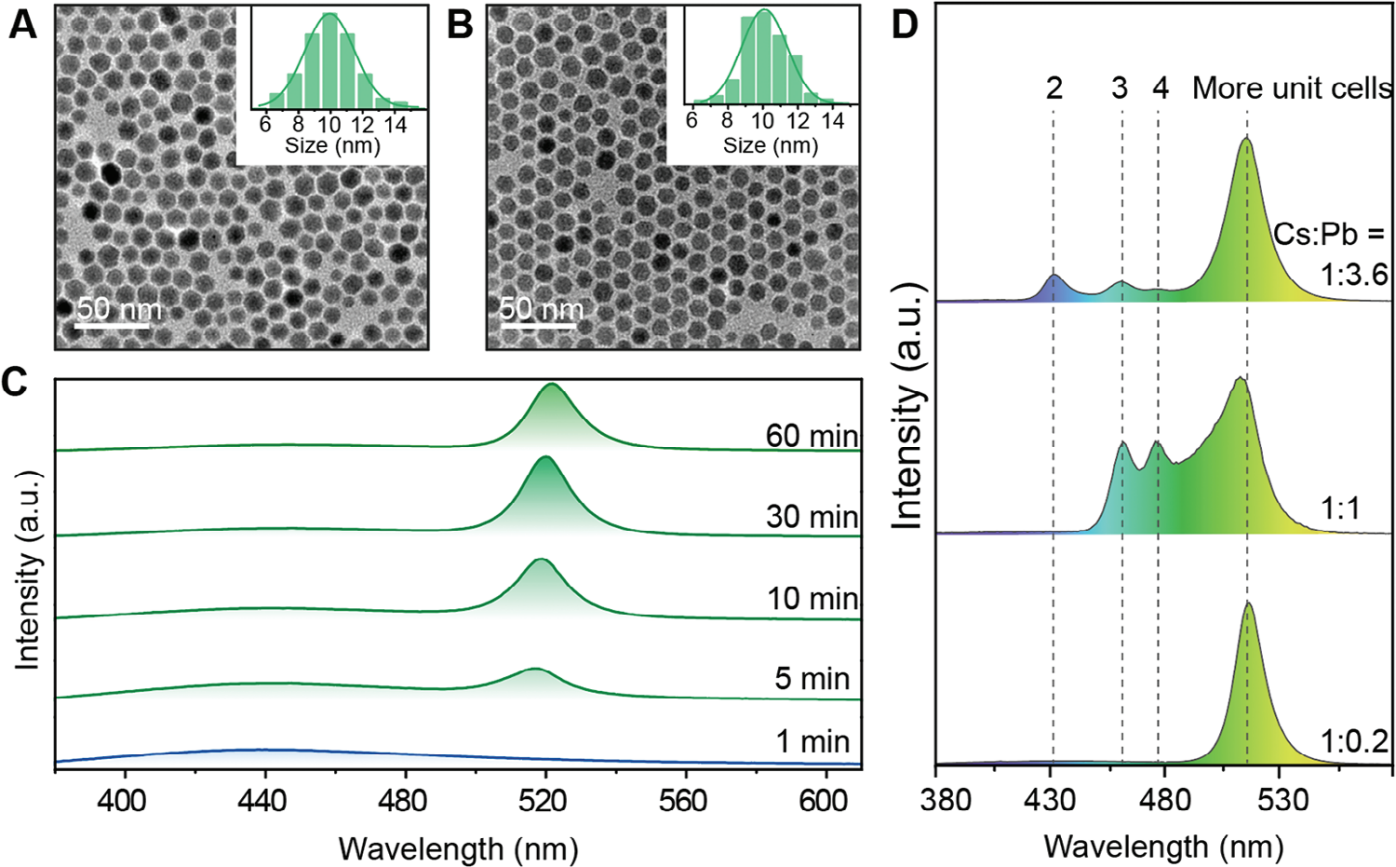
Lead halide perovskite synthesis and spectra tuning on a sub‐10 nm CsYb_2_F_7_ nanoplatform. A,B) Transmission electron micrographs of CsYb_2_F_7_ nanocrystals: pristine A) and post‐reaction B) with Pb^2+^ and Br^−^ ions. Insets show the corresponding size distributions. C) Emission spectra from CsYb_2_F_7_/CsPbBr_3_ at varying reaction durations, maintaining a 1:0.2 molar ratio of Cs^+^ to Pb^2+^. D) Emission spectra of the prepared CsYb_2_F_7_/CsPbBr_3_ products after 30 min of reaction at various Cs^+^‐to‐Pb^2+^ ratios (1:0.2, 1:1, and 1:3.6).

Alkali metal‐lanthanide fluorides are the most popular host materials for the development of highly stable luminescent nanomaterials.^[^ ^7^ ^]^ Using the co‐precipitation method,^[^ ^8^ ^]^ we synthesized CsYb_2_F_7_ nanoparticles at 290 °C, which serve as Cs^+^‐preconfined nanocrystals for perovskite synthesis. These nanocrystals have a diameter of about 10 nm and are linked to oleic acid (OA) ligands at the surface (**Figure** **2**A; Figure S1, and Table S1, Supporting Information). A solution containing PbBr_2_ dissolved in OA, oleylamine (OAm), and 1‐octadencene (ODE) was used for the subsequent growth of lead‐halide perovskites on the surface of as‐prepared CsYb_2_F_7_ nanocrystals. As the solution was injected at 180 °C for five minutes, green emission was observed under ultraviolet irradiation, which became brighter over time. Note that no additional Cs^+^ ions are introduced to the solvent, and the exclusive source of Cs^+^ ions is CsYb_2_F_7_ nanocrystals. The following chemical reaction equation might partially represent the synthesis process, as the inner part of CsYb_2_F_7_ is not participating in the reaction:

(1) $$3\mathrm{PbB}r_{2}+2\mathrm{CsY}b_{2}F_{7}\to 2\mathrm{CsPbB}r_{3}+4Yb^{3+}+14F^{-}+Pb^{2+}$$

While general perovskite synthesis can be completed rapidly, the development of green emissions on the CsYb_2_F_7_ nanoplatform takes much longer, with a plateau being reached after 30 min following injection (Figure 2B,C; Figure S2, Supporting Information). Moreover, the CsYb_2_F_7_ nanoplatform showed negligible changes in morphology after reaction (Figure 2B and Figure S3, Supporting Information). Although CsPbBr_3_ nanostructures formed on CsYb_2_F_7_ exhibited a spectral blue shift and had comparable lifetimes with standard CsPbBr_3_ quantum dots (Figure S4–S6, Supporting Information), no distinguishable X‐ray diffraction (XRD) peaks corresponding to CsPbBr_3_ were observed from the emitting nanoplatforms (Figure S7, Supporting Information). These results imply that the observed green emission is from few‐layer perovskites without notable Bragg diffraction. Meanwhile, Fourier‐transform infrared spectroscopy results confirmed the presence of oleamine on the surface of CsYb_2_F_7_/CsPbBr_3_ (Figure S8, Supporting Information). Intriguingly, when the initial molar ratio of Pb^2+^ to Cs^+^ confined in CsYb_2_F_7_ was increased, multiple emission bands with fixed peak positions emerged at shorter wavelength regions (Figure 2D). Since Br^−^ ions are the only halogens used, the emergence of these blue‐shifted emission peaks can be attributed to different degrees of exciton confinements.^[^ ^9^ ^]^ Therefore, the CsYb_2_F_7_ nanoplatform may provide a growth preference for lead‐halide perovskites that depends on the exposed facets.

**Figure 3:**
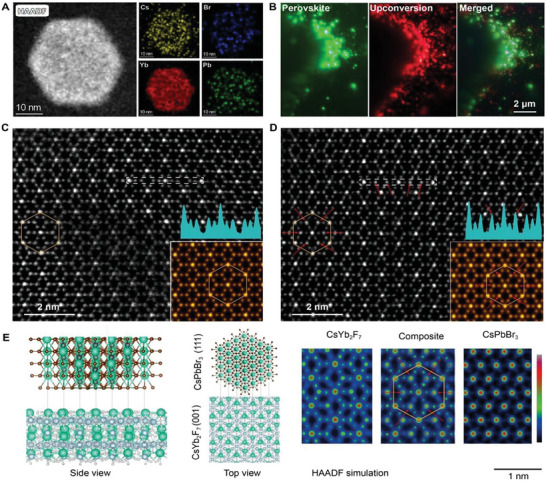
Structural investigation of lead‐halide perovskite nanostructures constructed on CsYb_2_F_7_ nanoplates. A) High‐angle annular dark field (HAADF) scanning transmission electron microscopy (STEM) image of a CsYb_2_F_7_ nanoplate after reaction and the corresponding elemental mapping (Cs^+^, Br^−^, Yb^3+^, and Pb^2+^). B) Optical micrographs of 2% Er^3+^‐doped CsYb_2_F_7_ nanoplates after reaction: under 365 nm excitation (left), 980 nm excitation (middle), and the merged image (right). C,D) HAADF‐STEM images of the CsYb_2_F_7_ platform and the CsYb_2_F_7_/CsPbBr_3_ composite. The inset shows the corresponding atomic arrangement from the inverse Fourier transform. E) Crystal structure of the CsYb_2_F_7_ nanoplatform and the CsPbBr_3_ perovskite in side view and top view. The right panel shows the HAADF simulation in top view for the (001) facet of CsYb_2_F_7_, the (111) facet of CsPbBr_3_, and their heterostructural composite.

Given that a specific crystal facet on a sub‐10 nm nanoplatform has a small surface area and is susceptible to drifting and decomposition under electron beams, we synthesized a larger plate‐like CsYb_2_F_7_:Er^3+^(2%) nanoplatform (30 nm in diameter) that predominantly exposes the (001) facet for perovskite synthesis and structural investigation (**Figure** **3**A and Figure S9, Supporting Information). By co‐doping Er^3+^ into CsYb_2_F_7_ nanoplates, strong red upconversion emission was observed in response to laser pumping at 980 nm, facilitating identification with an optical microscope. After the reaction, elements such as Cs, Yb, Pb, and Br can be identified in a single nanoplate (Figure 3A). The in‐situ growth of CsPbBr_3_ nanostructures on the nanoplate template was further confirmed by green emission after 365 nm excitation, which coincided with the red upconversion emission from nanoplates (Figure 3B).

We next examined the formation of perovskite nanostructures on the CsYb_2_F_7_ nanoplate by high‐resolution electron microscopy. High‐angle annular dark‐field (HAADF) imaging showed that crystal lattice sites on the (001) facet of the CsYb_2_F_7_ nanoplate became brighter after the reaction, indicating successful growth of Pb^2+^ ions (Figure 3C,D). It is noteworthy that the structure determined by electron microscopy agrees well with the modeling, showing that the (111) facet of the CsPbBr_3_ nanostructure is firmly docked to the (001) facet of the nanoplate (Figure 3E). CsPbBr_3_ reconstruction on the CsYb_2_F_7_ nanoplate is likely enabled by the minimal lattice mismatch (<5%) between these two facets.^[^ ^10^ ^]^ Extended X‐ray absorption fine structure (EXAFS) was further used to probe the coordination environment surrounding Yb^3+^ in CsYb_2_F_7_ and CsYb_2_F_7_/CsPbBr_3_ (Table S2 and Figure S10, Supporting Information). EXAFS spectra fitting results showed that the surface growth of CsPbBr_3_ on CsYb_2_F_7_ leads to a slight alteration in the coordination number of Yb^3+^ ions, increasing from 6.7 to 6.9. This suggests that the growth of surface perovskites has a limited impact on the inner coordination characteristics. It is conceivable that the different exposure of the Cs^+^ sublattice at each facet of the sub‐10 nm CsYb_2_F_7_ nanoplatform could lead to varying degrees of versatility in the nucleation and growth of perovskites, as reflected in the occurrence of multiple emission bands as a result of exciton confinements to different extents.

**Figure 4:**
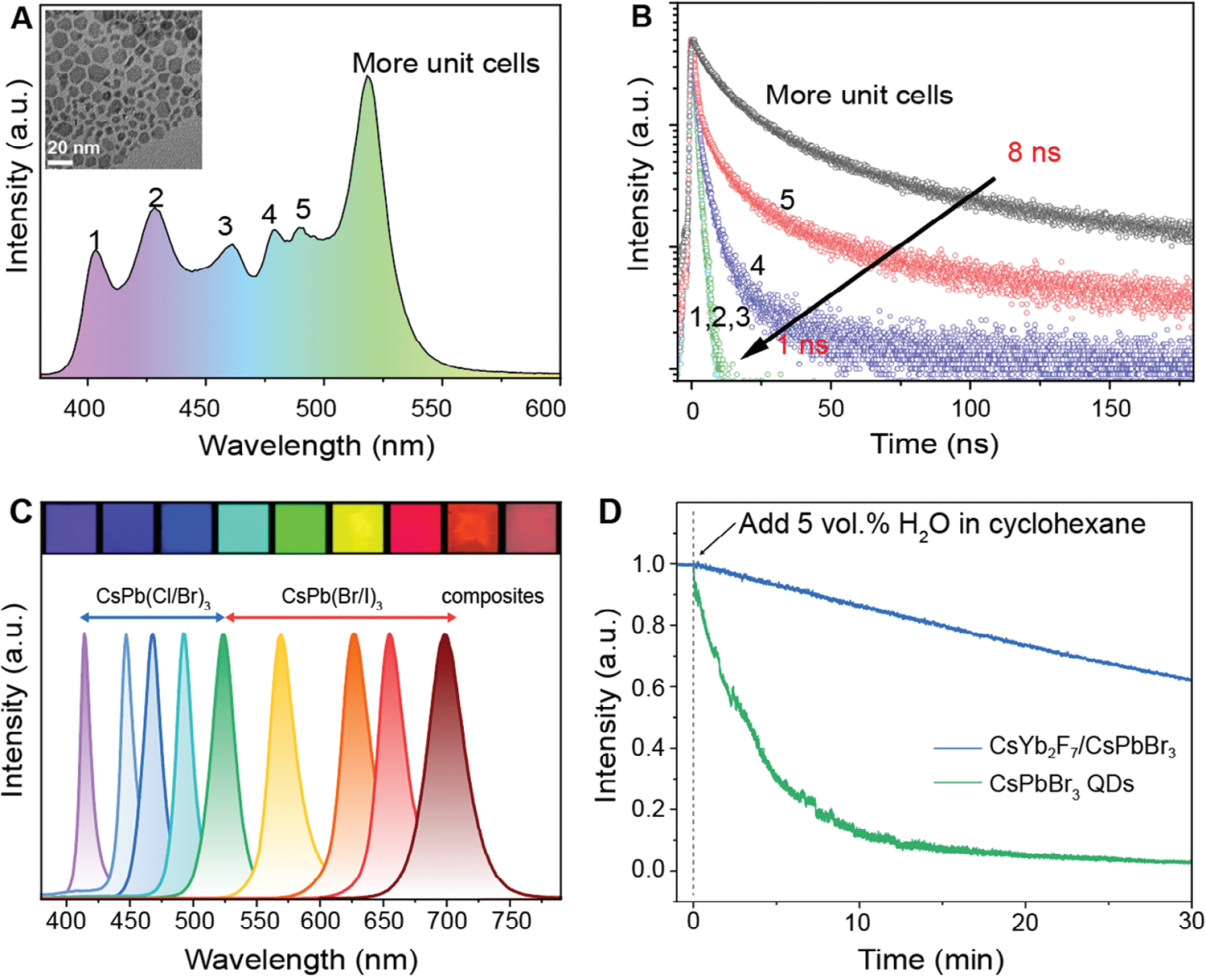
Cs^+^‐confined nanoplatform for the synthesis of lead halide perovskites with tunable luminescence. A) The emission spectrum of CsPbBr_3_ grown on CsYb_2_F_7_ nanoplates with low homogeneity, showing six transition bands. B) The variation of lifetime (from 8 to 1 ns) of the six transition emissions (from 517 to 402 nm) under 365 nm excitation. C) Emission spectra and corresponding photographs of perovskites prepared on nanorod templates with varied halogens. D) A stability comparison between conventional CsPbBr_3_ quantum dots and their counterparts developed on CsYb_2_F_7_ nanoplatforms.

To further explore the potential of this strategy in controlling perovskite growth and exciton confinement, we synthesized CsYb_2_F_7_ nanoplates with much lower homogeneity to expose as many crystal facets as possible for the subsequent preparation of lead‐halide perovskites. Compared with products obtained with uniform CsYb_2_F_7_ nanoparticles of size sub‐10 nm, two additional excitonic transition bands, blue‐shifted to 402 nm, were observed (**Figure** **4**A). It should be noted that the peak positions of these emission bands are consistent with those of reported low‐dimensional perovskite nanomaterials, suggesting success in molecular‐level engineering of perovskite nanostructures using the Cs^+^‐sublattice platform.^[^ ^11^ ^]^ Moreover, the strong quantum confinement enhances the interaction between the excitons and the surrounding environment, leading to increased nonradiative recombination and drastic luminescence lifetime decline from 8 to 1 ns (Figure 4B). We also found that this new strategy is applicable when switching from bromide to other halides. This allows lead‐halide perovskites with a wide spectral coverage from blue to deep red with no discernible difference for XRD after reaction using a homogeneous nanorod platform (Figure 4C; Figure S11–S15, Supporting Information). A high quantum yield of ≈86% was measured for as‐synthesized CsPbBr_3_ nanostructures (Figure S16, Supporting Information). Interestingly, the CsPbBr_3_ nanostructures prepared on the rigid Cs^+^‐sublattice nanoplatform show significantly improved water resistance compared to their counterparts prepared by conventional methods. When 5% water by volume was added to a cyclohexane solution of perovskite nanomaterials, more than 60% of the original intensity was retained after 30 min, while the CsPbBr_3_ QDs lost 90% of their original intensity in less than 10 min (Figure 4D).

**Figure 5:**
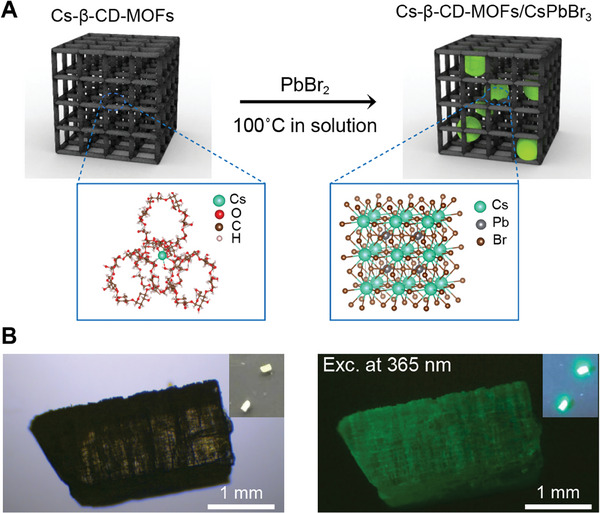
Conversion of Cs‐β‐CD‐MOFs to bright Cs‐β‐CD‐MOFs/CsPbBr_3_ hybrids. A) Schematic illustration of the Cs^+^‐MOF to MOF/Perovskite conversion process. The two 3D frames indicate the crystal structures of the Cs‐β‐CD‐MOFs (left) and Cs‐β‐CD‐MOFs/CsPbBr_3_ composite (right). B) Images of Cs‐β‐CD‐MOFs/CsPbBr_3_ composite under ambient light (left) and 365 nm UV stimulation (right).

Importantly, we demonstrated that in‐situ growth of highly luminous green‐emitting perovskites on a single crystal of a Cs‐based cyclodextrin (CD) metal‐organic framework (MOF) known as Cs‐β‐CD‐MOF. This achievement underscores the versatility and reliability of the approach for the development of hybrid functional nanosystems (**Figure** **5**).

## 3 Conclusion

Thus, by employing an ion‐enriched crystalline nanoplatform to control the synthesis kinetics precisely, we have developed a new strategy for preparing MHP nanostructures with tunable luminescence and improved stability. Our study indicates that the abundant crystal facet exposure of the Cs^+^‐preconfined nanoplatform can effectively impart different growth kinetics to the perovskite structure, resulting in varying degrees of exciton confinement and consequently multiple emission bands. While beam‐sensitive material‐friendly electron microscopy techniques are still needed to fully probe the nature of perovskite nucleation and growth on each facet, we believe that this strategy opens up a new avenue for creating high‐quality functional nanostructures. This development provides new opportunities for nanophotonics, quantum optics, energy conversion, and numerous other applications. We also speculate that this strategy could provide new insights for areas, such as atomic‐scale nanocrystal engineering and the control of excitonic transitions.

## Conflict of interest

The authors declare no conflict of interest.

4

## Supporting information

Supporting Information

## Data Availability

The data that support the findings of this study are available in the supplementary material of this article.
