# Supplementary material for: Promoted Growth and Multiband Emission in Heterostructured Perovskites Through Cs+‐Sublattice Interaction
Source: Adv Sci (Weinh). 2023 Nov 28;11(3):2306398. doi: 10.1002/advs.202306398 (PMC10797418; doi:10.1002/advs.202306398)
Supplement: Supplementary file 1 — Supporting Information [file ADVS-11-2306398-s001.pdf]

ADVANCED SCIENCE
Open Access

Supporting Information

for *Adv. Sci.*, DOI 10.1002/advs.202306398

Promoted Growth and Multiband Emission in Heterostructured Perovskites Through Cs^{+}-Sublattice Interaction

Lei Zhou, Liangliang Liang, Jiaye Chen, Xin Zhou, Lingmei Liu, Shibo Xi, Kian Ping Loh, Yu Han, Qian He* and Xiaogang Liu**

Supporting Information for

Promoted Growth and Multiband Emission in Heterostructured Perovskites through Cs^+-Sublattice Interaction

Lei Zhou [a,b], Liangliang Liang [a,i]*, Jiaye Chen [a], Xin Zhou [c], Lingmei Liu [d], Shibo Xi [e], Kian Ping Loh [a], Yu Han [f], Qian He [c]*, Xiaogang Liu [a,g,h]*

*Corresponding author. E-mail: e-mail: ifellliang@xmu.edu.cn, mseheq@nus.edu.sg, chmlx@nus.edu.sg

WILEY-VCH

Materials and methods

Chemicals. The following chemicals were purchased from Sigma-Aldrich: sodium hydroxide (NaOH; >98%), cesium hydroxide monohydrate ($CsOH{\cdot}H_2O$; >90%), ytterbium acetate hydrate ($Yb(CH_3CO_2)_3{\cdot}xH_2O$; 99.9%), ammonium fluoride (NH_4F; >98%), oleylamine (OAm; technical grade 70%), oleic acid (OA; technical grade 90%) and 1-octadecene (ODE; technical grade 90%). Lead iodide (PbI_2; 99.9%), lead bromide ($PbBr_2$; 99%), lead chloride ($PbCl_2$; 99.99%), β-Cyclodextrin (β-CD), dichloromethane (DCM) and polyethylene glycol 20000 (PEG20000) were purchased from Aladdin, Co., Ltd (Shanghai, China). All chemicals were used as starting materials without further purification.

Preparation of $CsYb_2F_7$ nanocrystals. $CsYb_2F_7$ nanocrystals were synthesized using a modified method with a Cs: Yb: F molar ratio of 1.2:2:7 [S1]. First, an aqueous solution of $Yb(CH_3COO)_3$ (0.8 mmol, 4.0 ml) was mixed with oleic acid (4 mL) and 1-octadecene (6 mL) in a round-bottomed flask. The mixture was then heated at 150 °C for 1 hour to remove water. After cooling to 50 °C, a methanol solution of cesium hydroxide (0.48 mmol, 0.96 mL) and ammonium fluoride (2.8 mmol, 7 mL) was added and stirred for 30 minutes at 50 °C, followed by heating at 100 °C for another 30 minutes. Finally, the mixture was heated to 290 °C and kept under nitrogen for 2 hours. After cooling to room temperature, nanocrystals were washed three times with ethanol and redispersed in 4 mL of cyclohexane for $CsYb_2F_7$ /$CsPbX_3$ synthesis.

Preparation of $CsYb_2F_7$ nanoplates. $CsYb_2F_7$ nanoplates were synthesized by the same procedure except for the ratio of Cs:Yb: F being 2:5:8.

Preparation of irregularly shaped $CsYb_2F_7$ nanocrystals. $CsYb_2F_7$ nanoplates were synthesized by the same procedure but with accelerated heating and cooling.

Synthesis of $CsPbBr_3$ nanomaterials. Cesium lead bromide ($CsPbBr_3$) nanomaterials were synthesized by the hot-injection method [S2]. In the experiment, 0.38 mmol of lead bromide ($PbBr_2$) was dissolved in a mixture of oleic acid (1.0 mL), oleylamine (1.0 mL), and octadecene (15 mL) in a round-bottomed flask. The mixture was heated to 100 °C for 0.5 hours under vacuum to remove moisture and continuously introduced nitrogen gas. The mixture was then increased to 160 °C until the $PbBr_2$ was completely dissolved. A hot solution of cesium oleate (1 mL, 0.1514 M) was quickly injected into the mixture at 180 °C. After 5 seconds, the flask was transferred to an ice bath. $CsPbBr_3$ quantum dots were

WILEY-VCH

obtained by centrifugation at 13,000 rpm for 10 minutes and stored in 4 mL of cyclohexane. Mixed-halide perovskite QDs were synthesized by varying the halide composition.

Synthesis of $CsYb_2F_7/CsPbX_3$ nanostructures. Cesium ytterbium fluoride/cesium lead bromide ($CsYb_2F_7/CsPbX_3$) nanomaterials were synthesized using a modified hot-injection method [S2]. In the experiment, 0.02-0.36 mmol of lead bromide ($PbBr_2$), 1.0 mL of oleic acid (OA), 1.0 mL of oleylamine (OAm), and 10 mL of octadecene (ODE) were added to a three-neck round-bottomed flask. The mixture was heated under vigorous stirring in vacuum to 100 °C for 0.5 hours; then, the moisture residue was removed by purging with nitrogen. The mixture was heated to 180 °C until the $PbBr_2$ was dissolved. A solution of $CsYb_2F_7$ nanocrystals in cyclohexane (1.0 mL, 0.1M) was quickly injected and allowed to undergo a 30-min reaction before cooling in an ice-water bath. The resulting solution was transferred to a mixed solvent of octadecene (3.5 mL), oleic acid (0.35 mL), and oleylamine (0.35 mL) and stirred at room temperature for different reaction times (10 seconds, 1 minute, 5 minutes, 10 minutes, 30 minutes, and 1 hour). The resulting samples were centrifuged at 6,000 rpm for 5 minutes, and the supernatant was washed twice with methyl acetate. Finally, the $CsYb_2F_7/CsPbBr_3$ nanomaterials were obtained by centrifugation at 13,000 rpm for 10 minutes and stored in 4 mL of cyclohexane. Different $CsYb_2F_7/CsPbX_3$ (X=Cl, Cl/Br, and Br/I) samples were synthesized following the same procedure.

Synthesis of nanorods. Cesium-rare earth-fluoride nanorod hosts were synthesized by a modified hot-injection method. In a typical experiment, $Y(CH_3CO_2)_3$ (1.0 mL, 0.2 mmol) and $Yb(CH_3CO_2)_3$ (1.0 mL, 0.2 mmol) were dissolved in a mixture of ODE (6.0 mL) and OA (4.0 mL) in a round-bottomed flask equipped with a temperature sensor. The mixture was stirred at 150 °C for 40 minutes to remove moisture. After cooling to 50 °C, a methanol solution of NH_4F (4.0 mL, 1.6 mmol), NaOH (1 mL, 0.5 mmol), and CsOH (1 mL, 0.5 mmol) was added and stirred for 30 minutes. The reaction mixture was then vacuumed at 105 °C for 15 minutes and heated to 290 °C for 2.5 hours under nitrogen. After cooling to room temperature, the resulting nanorods were isolated by washing with ethanol and finally dispersed in 4.0 mL of cyclohexane. The Na: Cs: Y: Yb: F ratio was set at 2.5:2.5:1:1:8.

Synthesis of nanorod composites with different halides. The cesium-rare earth-fluoride nanorod hybrid nanostructures were synthesized by the same injection procedure. In a typical experiment, PbX_2 (X=Cl, Br and I, 0.2 mmol), OA (1.0 mL), OAm (1.0 mL), and ODE (10 mL) were added to a two-neck round-bottomed flask. The reaction mixture was heated to 100 °C with vigorous stirring under vacuum

for 30 min. Afterward, the flask was purged with N_2 and subjected to vacuum again to remove any remaining moisture residue by applying N_2 and vacuum several times. The temperature was then increased to 180 °C until the PbX_2 precursors were completely dissolved. At this point, a quick injection of the cyclohexane precursor solution (1 mL) was added to the mixture. After 30 min of reaction, the flask was transferred to an ice bath. The composite was obtained by centrifugation at 13000 rpm for 10 minutes, then washed twice with methyl acetate and stored in 4 mL of cyclohexane.

Synthesis of Cs-β-CD-MOFs. A modified methanol vapor diffusion method was used to grow Cs-β-CD-MOFs crystals [S3]. First, β-cyclodextrin (0.1418 g, 0.125 mmol) and $CsOH·H_2O$ (0.1894 g, 1 mmol) were dissolved in water (5 mL). This solution was then filtered and placed in a small container, along with 0.5 mL of methanol. The small container was further stored inside a larger container containing 60 mL of methanol. Over a 12-h period at 50 °C, methanol vapor diffused into the water solution, facilitating the growth of Cs-β-CD-MOFs crystals. The resulting crystals were collected by adding a 5 mL methanol solution containing 60 mg of PEG20000 and allowing it to incubate overnight. The obtained products were then washed with ethanol and dichloromethane and dried at 50 °C under vacuum.

Synthesis of Cs-β-CD-MOFs/$CsPbBr_3$ hybrids. Cs-based metal-organic framework (MOF) perovskite composites were synthesized using a liquid-phase heating reaction. In a typical experiment, $PbBr_2$ (0.2 mmol), OA (1.0 mL), OAm (1.0 mL), and ODE (10 mL) were added to a two-neck round-bottom flask. The reaction mixture was heated to 100 °C with vigorous stirring. Afterward, the synthesized Cs-β-CD-MOFs were added to the mixture. After 10 min of reaction, a bright Cs-β-CD-MOFs/$CsPbBr_3$ composite was obtained and collected by filtration.

Characterizations.

The size and morphology of the nanocrystals prepared were characterized using a JEOL-1400 transmission electron microscope (TEM) operating at an acceleration voltage of 100 kV and a JSM-6701F field emission scanning electron microscope (FESEM) with accelerating voltage adjustable from 0.5 to 30 kV. Powder X-ray diffraction patterns were obtained using a Bruker D8 X-ray diffractometer with CuKa radiation (40 kV, 40 mA, λ=1.5404 Å). Luminescence spectra of samples dispersed in cyclohexane were recorded using an Edinburgh FLS1000 spectrofluorometer with a photomultiplier. The lifetimes of the $CsYb_2F_7$/$CsPbBr_3$ composites were measured after excitation by a nanosecond-pulsed (371 nm) LED source. Photons were detected using a photomultiplier tube coupled to a HORIBA

WILEY-VCH

spectrometer. UV-visible absorption spectra were obtained using an Agilent Cary 3500 UV-Vis-NIR spectrometer. Fourier transform infrared (FT-IR) spectra were measured by a PerkinElmer FT-IR Spectrometer (Spectrum 100) from 4000 cm^{-1} to 500 cm^{-1}. Scanning transmission electron microscopy (STEM) with ASCOR aberration corrector was performed using a JEOL ARM200F microscope operating at an 80 kV accelerating voltage. X-ray photoelectron spectroscopy (XPS) was conducted using the Phoibos 150 NAP-XPS package equipped with a twin anode X-ray source (SPECS XR50, Al Ka, hυ = 1486.6 eV; Mg Ka, hυ = 1253.6 eV) and a base pressure below 5×10^{-10} mbar. Synchrotron radiation characterizations for the Yb-L_3 edge were carried out at room temperature in the XAFCA beamline of the Singapore Synchrotron Light Source (SSLS) operated at 0.7 GeV and a current of 150 mA. Data was collected with a monochromator using two flat Si (111) crystals in focusing mode.

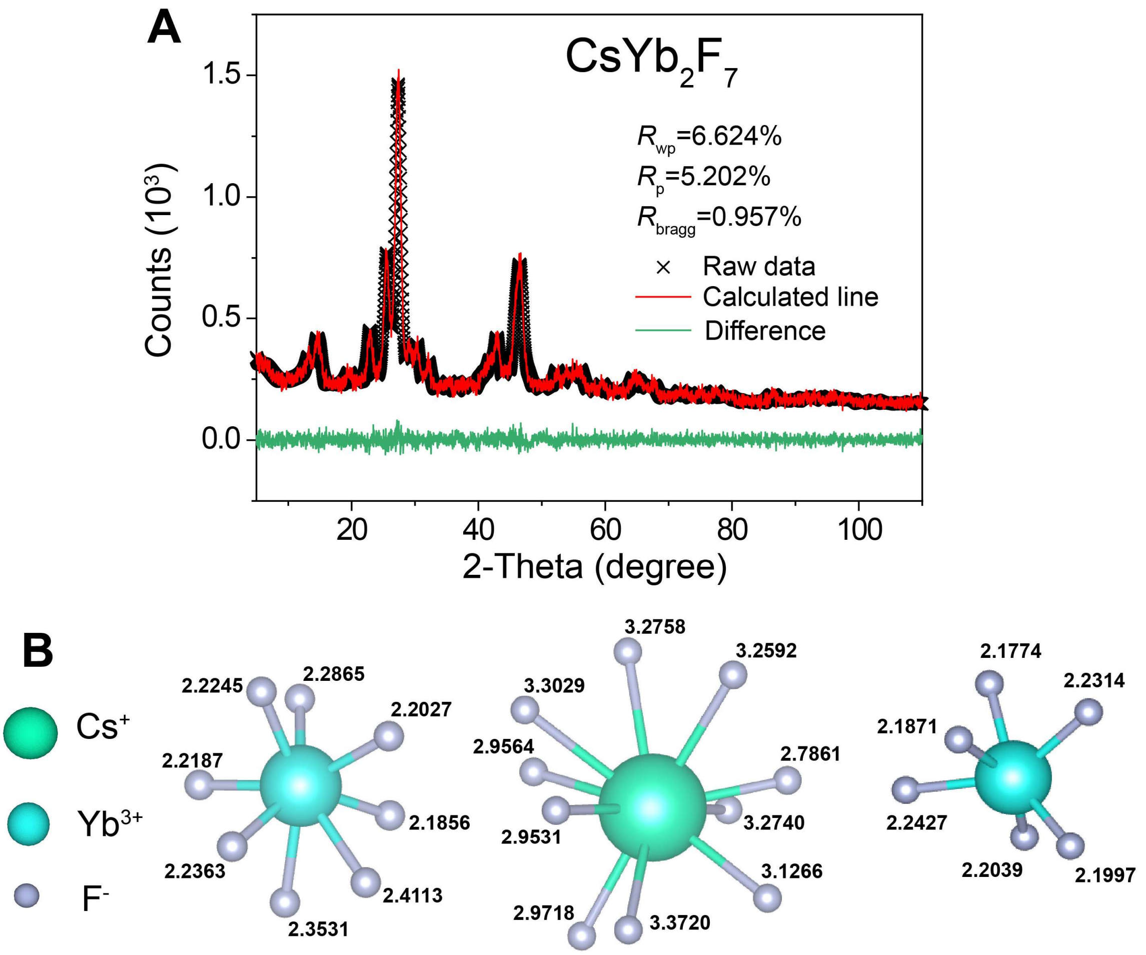

Figure S1. (**A**) X-ray diffraction (XRD) patterns and structural refinement of the prepared host. All peaks are well indexed in accordance with the simulated monoclinic $CsYb_2F_7$ structure. (**B**) Bond distances labeled for Yb^{3+} and Cs^+, with average values of 2.2648 Å (CN=8), 3.1278 Å (CN=10), and 2.2070 Å (CN=6), respectively. CN stands for the coordination number.

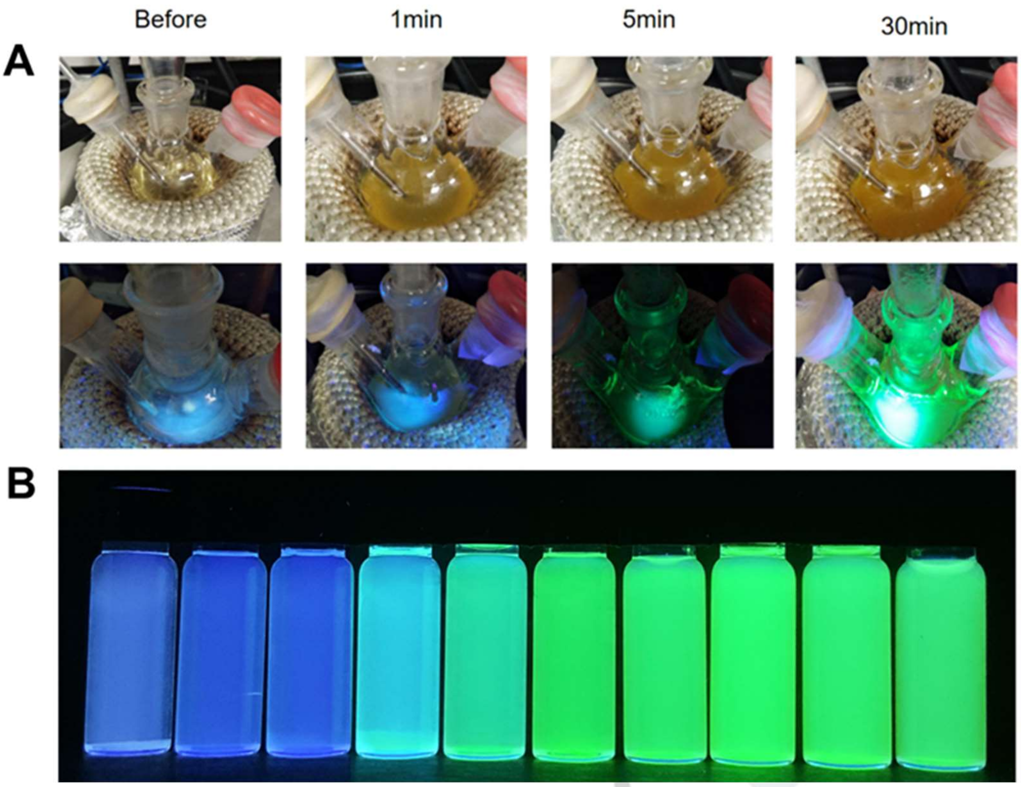

Figure S2. (**A**) Digital photographs taken under natural light and 365 nm light excitation. (**B**) Resulting products under 365 nm excitation at different reaction times (10 s, 1 min, 5 min, 10 min, 30 min, 1 h, 1.5 h, 2 h, and 2.5 h).

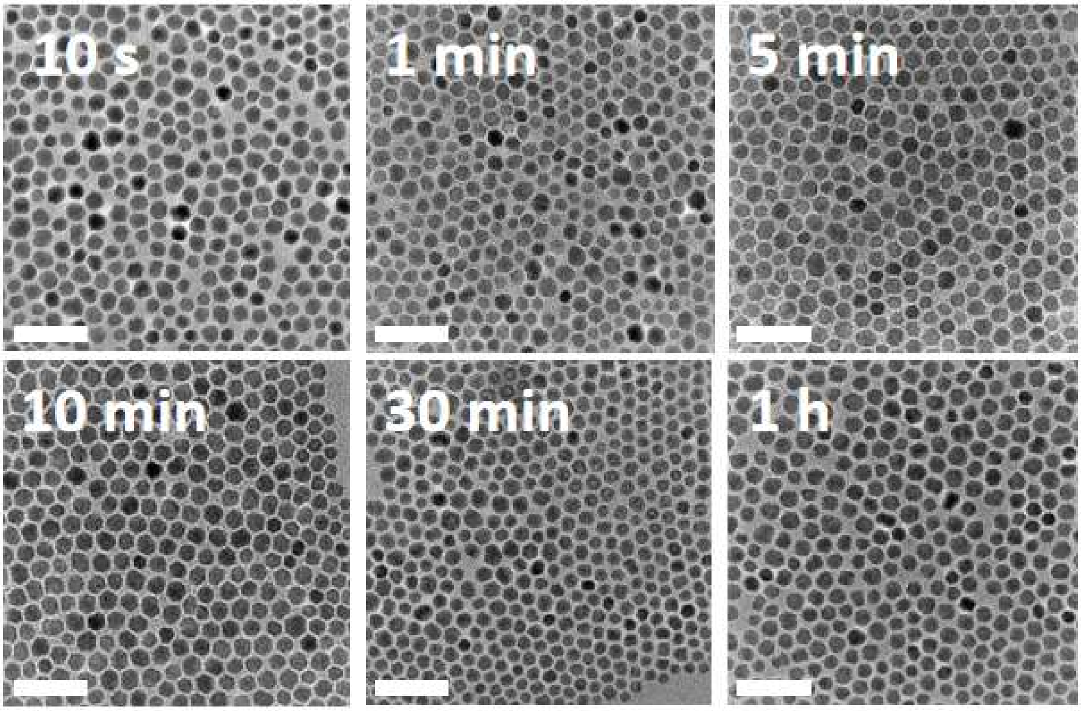

Figure S3. Transmission electron microscopy images of the as-prepared $CsYb_2F_7$/$CsPbBr_3$ at different reaction times (from 10 s to 1 h), showing a persistent size distribution of about 10 nm in diameter.

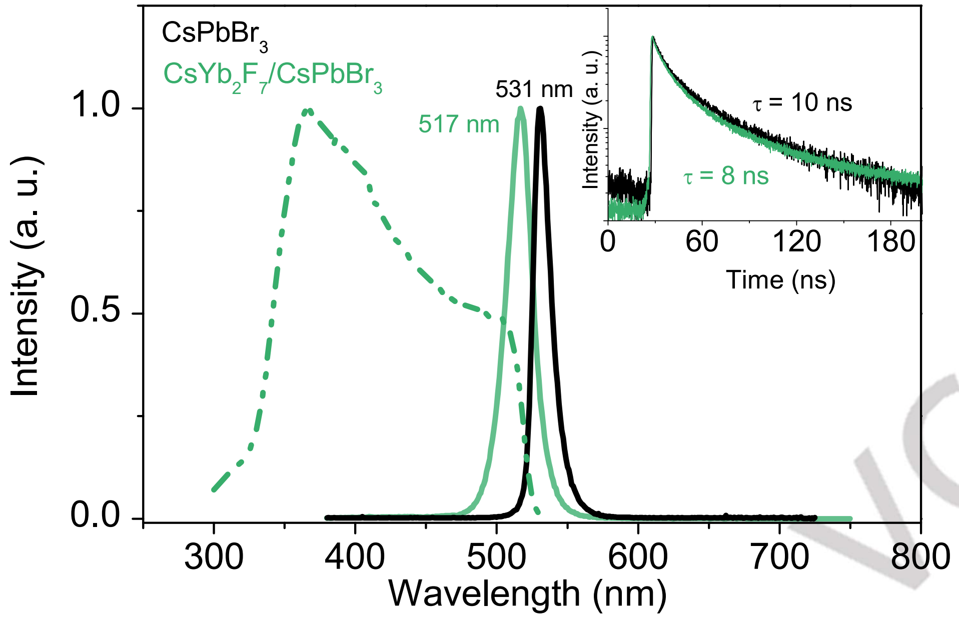

Figure S4. Excitation (λ_{em}=550 nm) and emission spectra (λ_{ex}=365 nm) of the as-prepared $CsYb_2F_7/CsPbBr_3$ nanostructures and $CsPbBr_3$ (black line, λ_{ex}=365 nm). The inset shows the luminescence decay curves of the prepared nanostructure (green line, λ_{ex}=405 nm, λ_{em}=517 nm) and $CsPbBr_3$ (black line, λ_{ex}=405 nm, λ_{em}=531 nm).

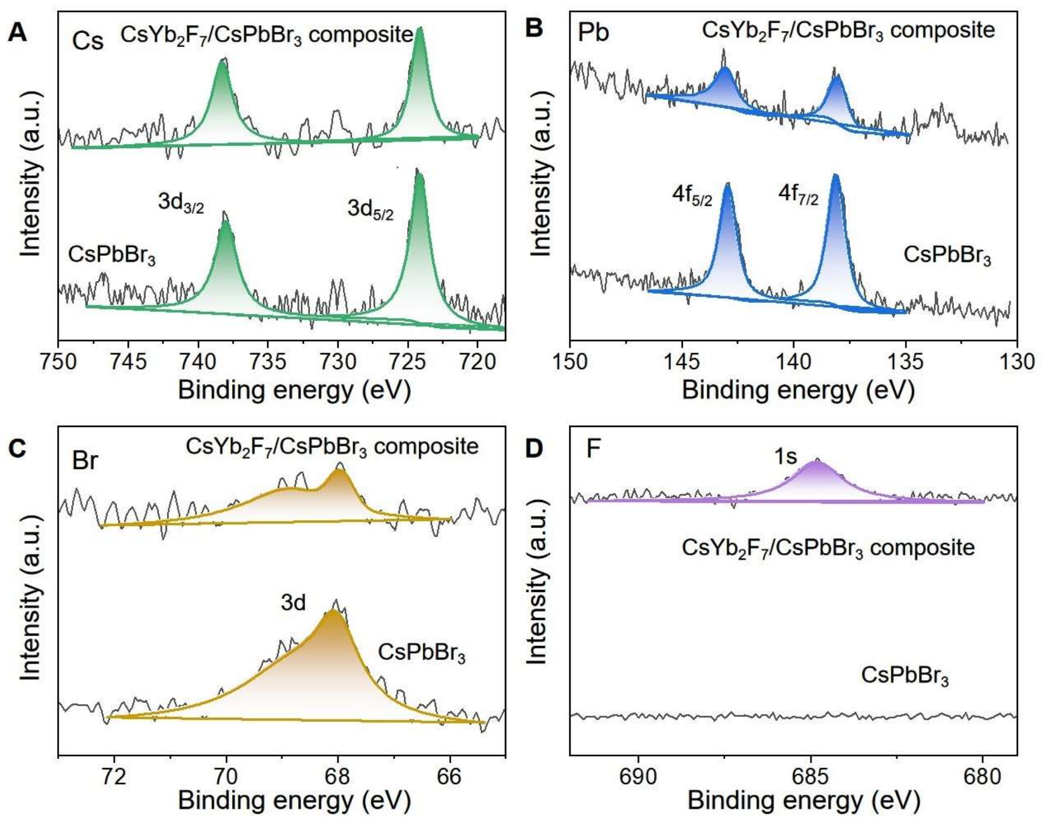

Figure S5. High-resolution X-ray photoelectron spectroscopy analysis for the elements (**A**) Cs (**B**) Pb (**C**) Br, and (**D**) F of $CsYb_2F_7/CsPbBr_3$ and $CsPbBr_3$ nanocrystals.

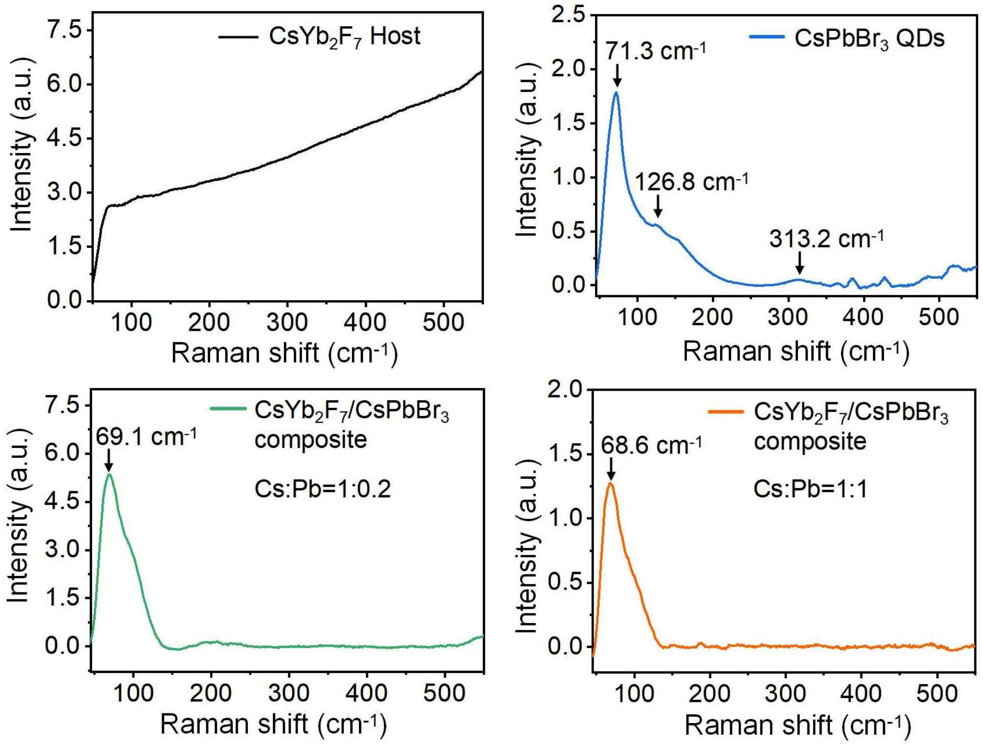

Figure S6. Raman spectra of host samples and $CsYb_2F_7/CsPbBr_3$ nanostructures, which were prepared with varying ratios of Cs to Pb, indicating that the hybrid nanostructures exhibit a distinctive Raman peak at approximately 70 cm^{-1}, similar to that of $CsPbBr_3$ QDs.

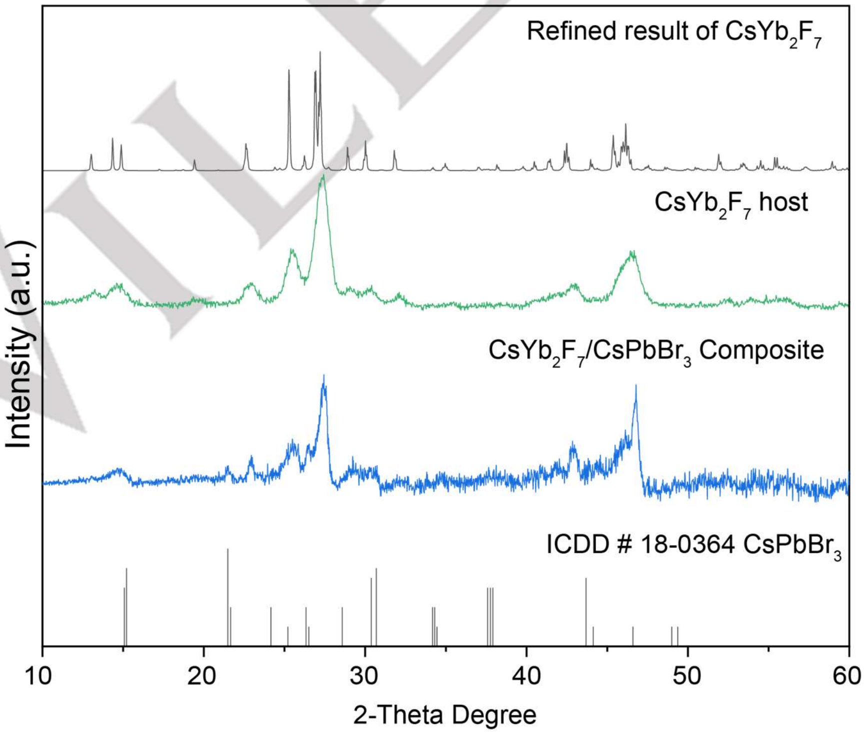

Figure S7. X-ray diffraction patterns of the sub-10 nm nanoplatform and the prepared $CsYb_2F_7/CsPbBr_3$ composites.

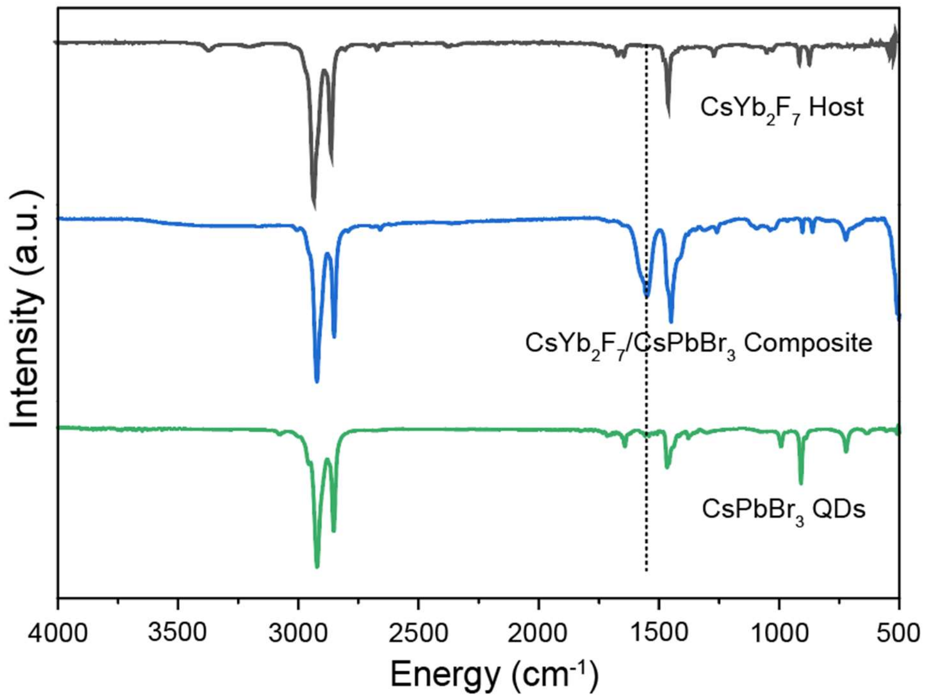

Figure S8. Fourier-transform infrared spectra of the $CsYb_2F_7$ nanoplatform, $CsPbBr_3$ QDs, and the prepared $CsYb_2F_7/CsPbBr_3$ composite.

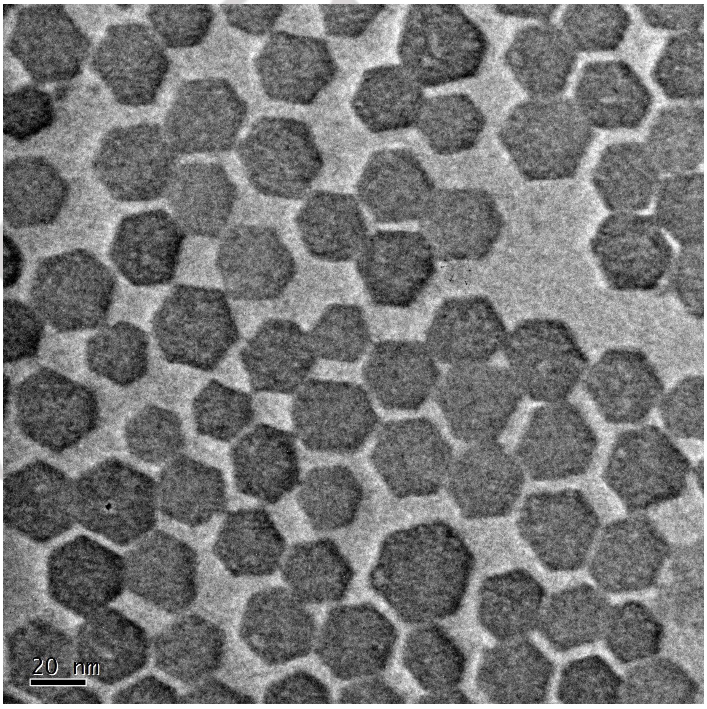

Figure S9. Transmission electron microscopy imaging of the as-prepared $CsYb_2F_7/CsPbBr_3$ nanoplates.

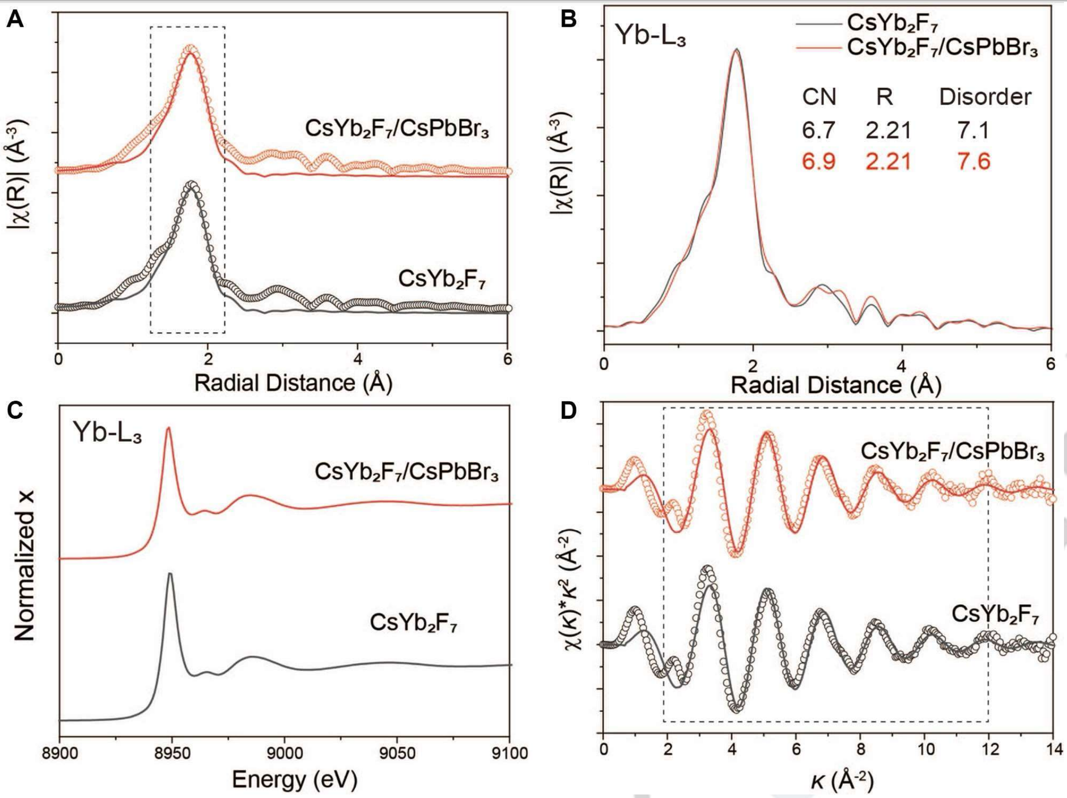

Figure S10. Synchrotron radiation-based XAFS structural characterization of $CsYb_2F_7$ and $CsYb_2F_7/CsPbBr_3$. (**A**) Experimental (dots) and the fitting (solid lines) results of Fourier transforms of Yb-L_3 edge k^2-weighted EXAFS spectra. The window represents the fitting range (1.2 ≤ R ≤ 2.2 Å). (**B**) Comparison of Fourier transforms of Yb-L_3 edge k^2-weighted EXAFS experimental spectra. (**C**) Yb-L_3 edge X-ray absorption near edge spectroscopy. (**D**) Yb-L_3 edge k^2-weighted EXAFS experimental (dots) and the fitting (solid lines) results. The window represents the fitting range (1.9 ≤ k ≤ 12.0 $Å^{-1}$).

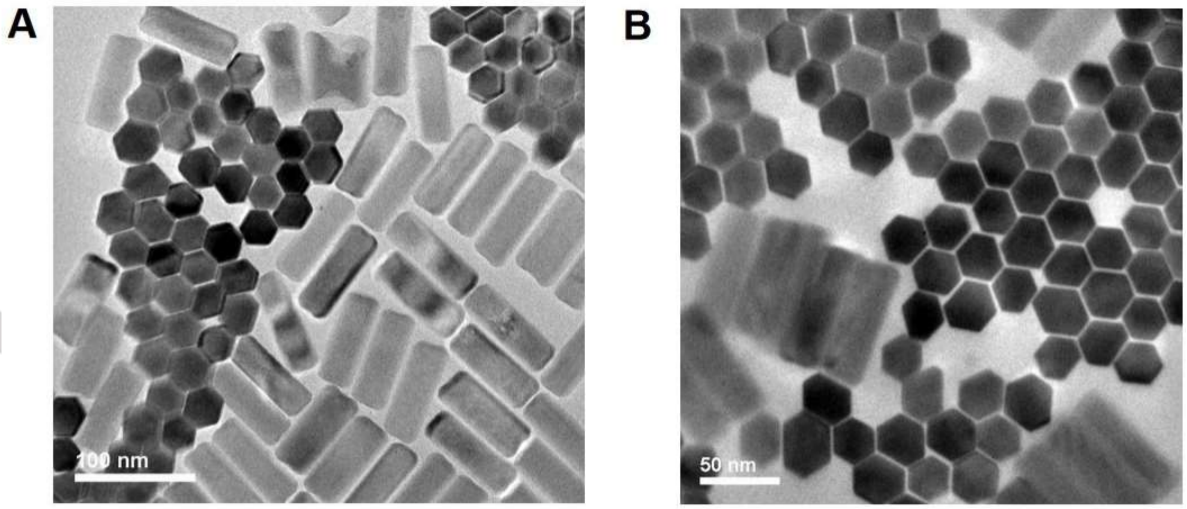

Figure S11. TEM images of (**A**) the cesium-rare earth-fluoride nanorods host and (**B**) the corresponding $CsPbBr_3$ hybrid nanostructures.

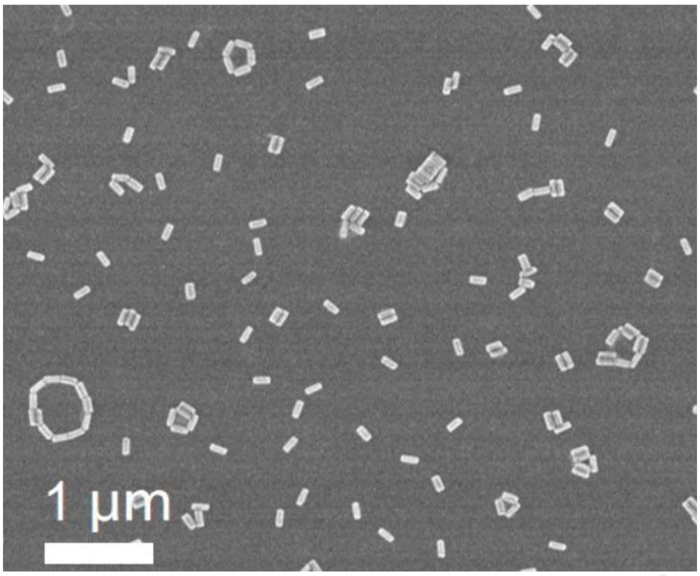

Figure S12. Field-emission scanning electron microscopy image of the cesium rare-earth fluoride nanorod/perovskite hybrids.

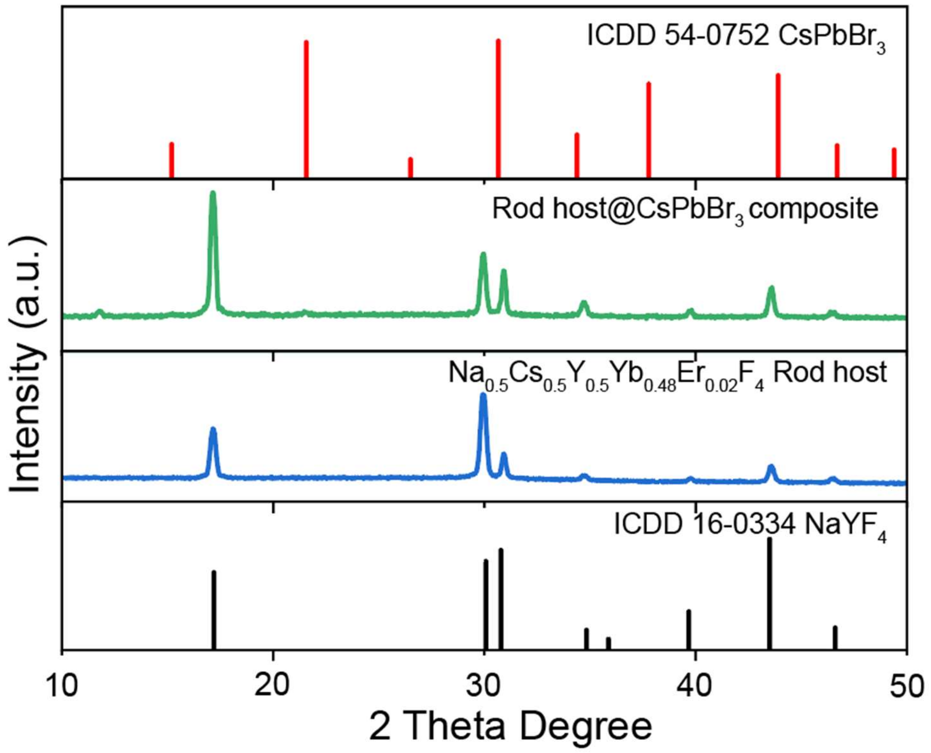

Figure S13. X-ray diffraction patterns of the cesium-rare earth-fluoride nanorod platform and the corresponding hybrid nanostructures.

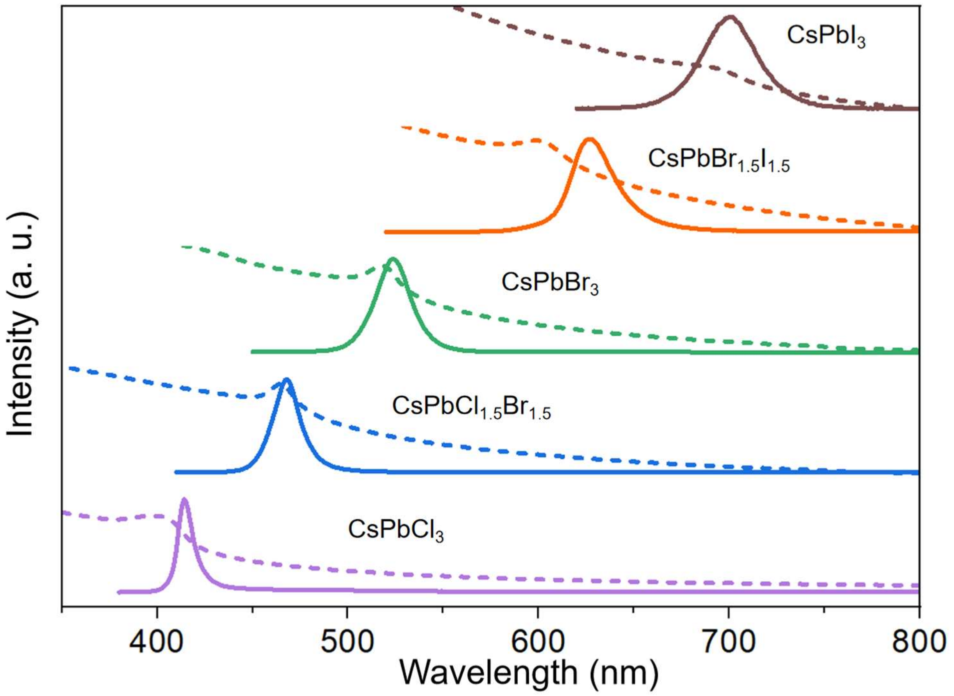

Figure S14. Absorption and emission spectra of $CsYb_2F_7/CsPbX_3$ hybrid nanostructures with different halide compositions (X= Cl, Br, and I).

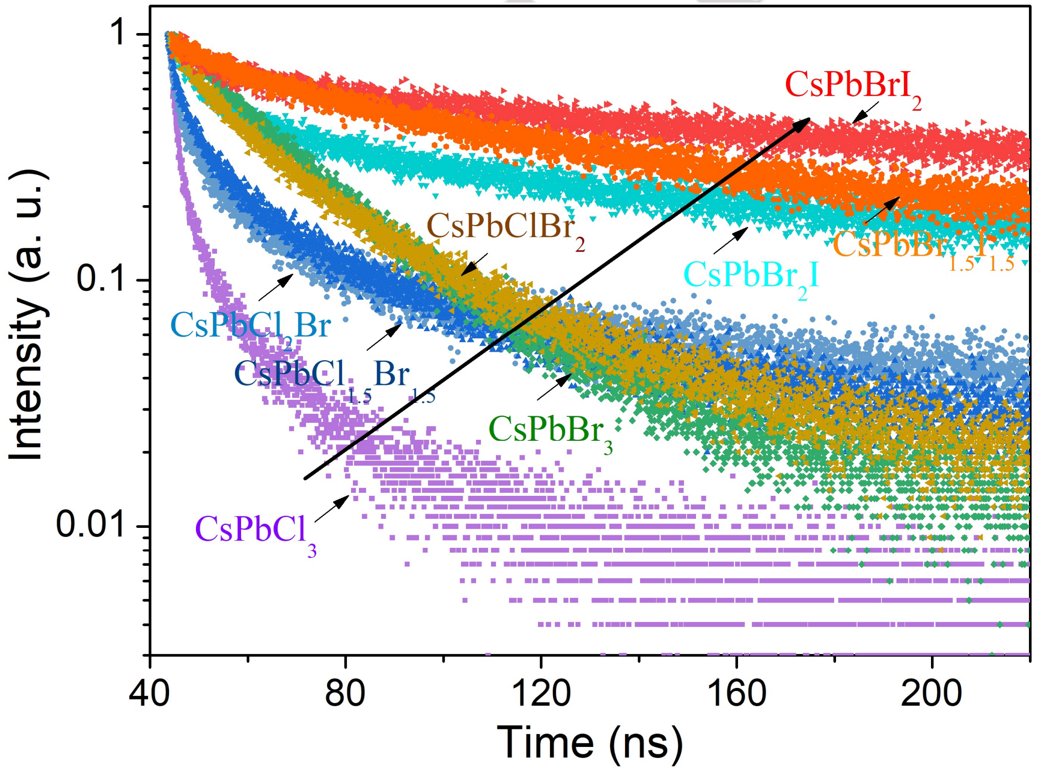

Figure S15. Time-resolved photoluminescence measurements of $CsYb_2F_7/CsPbX_3$ hybrid nanostructures with varying halide content ($CsPbCl_3$, $CsPbCl_2Br$, $CsPbCl_{1.5}Br_{1.5}$, $CsPbClBr_2$, $CsPbBr_3$, $CsPbBr_2I_1$, $CsPbBr_{1.5}I_{1.5}$ and $CsPbBrI_2$).

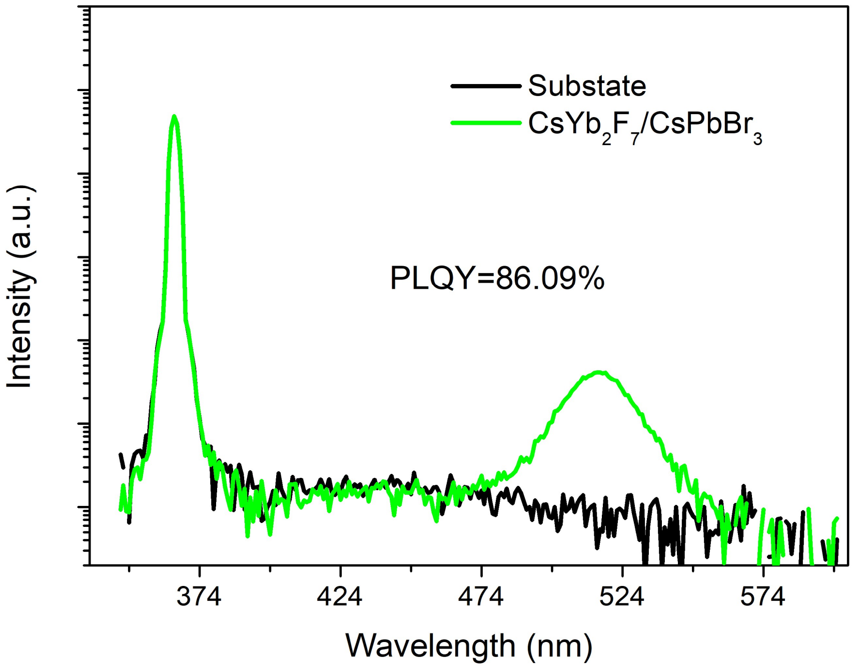

Figure S16. The quantum yield of the as-prepared $CsYb_2F_7/CsPbBr_3$ nanostructures under 365 nm excitation.

Table S1. Main parameters of the refined $CsYb_2F_7$ nanocrystals.

Compound	$CsYb_2F_7$ NCs
Space group	$P112_1/b$ (14)
a	15.5820 Å
b	15.5491 Å
c	12.2892 Å
α	90°
β	90°
γ	110.89°
V= Å^3	2581.4313
Z	16
Rwp	6.624%
Rp	5.202%
Rbragg	0.957%

Table S2. The fitting parameters of Yb-L_3 edge EXAFS.

Nanocrystal	R (Å)	CN	σ^2(×10^{-3}Å^2)	ΔE_0 (eV)	R factor (%)
$CsYb_2F_7$	2.21 ± 0.01	6.7 ± 0.6	7.1 ± 1.7	1.8 ± 0.9	0.79
$CsYb_2F_7$/$CsPbBr_3$	2.21 ± 0.01	6.9 ± 0.6	7.6 ± 1.9	1.7 ± 1.0	0.60

References

[S1] F. Wang, Y. Han, C. S. Lim, Y. H. Lu, J. Wang, J. Xu, H. Y. Chen, C. Zhang, M. H. Hong, X. G. Liu, Nature **2010**, 463, 1061.
[S2] A. Swarnkar, A. R. Marshall, E. M. Sanehira, B. D. Chernomordik, D. T. Moore, J. A. Christians, T. Chakrabarti, J. M. Luther. Science **2016**, 354, 92.
[S3] W. Jiang, H. Liu, Q. Liao, T. Tang, J. Liu, Z. Liu, L. Xie, J. Yan, Polyhedron **2021**, 196, 114983.

WILEY-VCH
